# The Fight against Plant-Parasitic Nematodes: Current Status of Bacterial and Fungal Biocontrol Agents

**DOI:** 10.3390/pathogens11101178

**Published:** 2022-10-13

**Authors:** David Pires, Cláudia S. L. Vicente, Esther Menéndez, Jorge M. S. Faria, Leidy Rusinque, Maria J. Camacho, Maria L. Inácio

**Affiliations:** 1 Instituto Nacional de Investigação Agrária e Veterinária (INIAV, I.P.), Av. da República, 2780-159 Oeiras, Portugal; 2 Mediterranean Institute for Agriculture, Environment and Development (MED) & Global Change and Sustainability Institute (CHANGE), Institute for Advanced Studies and Research, University of Évora, Pólo da Mitra, Apartado 94, 7006-554 Évora, Portugal; 3 Department of Microbiology and Genetics, Institute for Agribiotechnology Research (CIALE), Universidad de Salamanca, 37007 Salamanca, Spain; 4 GREEN-IT Bioresources for Sustainability, Instituto de Tecnologia Química e Biológica, Universidade Nova de Lisboa (ITQB NOVA), Av. da República, 2780-157 Oeiras, Portugal

**Keywords:** bacteria, biological control, bionematicides, cyst nematodes, nematophagous fungi, pinewood nematode, root-knot nematodes, root-lesion nematodes

## Abstract

Plant-parasitic nematodes (PPNs) are among the most notorious and underrated threats to food security and plant health worldwide, compromising crop yields and causing billions of dollars of losses annually. Chemical control strategies rely heavily on synthetic chemical nematicides to reduce PPN population densities, but their use is being progressively restricted due to environmental and human health concerns, so alternative control methods are urgently needed. Here, we review the potential of bacterial and fungal agents to suppress the most important PPNs, namely *Aphelenchoides besseyi*, *Bursaphelenchus xylophilus*, *Ditylenchus dipsaci*, *Globodera* spp., *Heterodera* spp., *Meloidogyne* spp., *Nacobbus aberrans*, *Pratylenchus* spp., *Radopholus similis*, *Rotylenchulus reniformis*, and *Xiphinema index*.

## 1. Introduction

Nematodes are non-segmented invertebrates and are by far the most abundant animals on Earth [1], accounting for an estimated four-fifths of all animals of the terrestrial biosphere [2]. Among soil-dwelling nematodes, some have crucial ecological niches in the soil food web, regulating carbon and recycling nutrients (such as nitrogen, increasing its availability to plants) [3,4], while others are considered a phytosanitary risk.

Plant-parasitic nematodes (PPNs) pose a big threat to food security and plant health, with estimated annual global economic losses of USD 173 billion [5]. The Commission Implementing Regulation (EU) 2019/2072 lists 15 nematode species, 10 of which do not occur in the Schengen territory and 5 do [6]. The European and Mediterranean Plant Protection Organization (EPPO) recommends EU member states to regulate the following nematodes as quarantine pests: *Aphelenchoides besseyi*, *Bursaphelenchus xylophilus*, *Ditylenchus dipsaci*, *Globodera pallida*, *G. rostochiensis*, *Heterodera glycines*, *Meloidogyne chitwoodi*, *M. enterolobii*, *M. fallax*, *M. mali*, *Radopholus similis*, and *Xiphinema rivesi* [7]. The Asia and Pacific Plant Protection Commission records nine A2 PPNs (pests that are present but not widely distributed) [8], whereas the Inter-African Phytosanitary Council only lists two [9]. In the United States, the Animal and Plant Health Inspection Service (USDA APHIS) includes over 60 PPNs in their Regulated Plant Pests table [10].

Symptoms of PPN damage to crop development are mostly non-specific and are often mistaken for abiotic stress, and thus PPN infection frequently goes untreated. This can lead to extreme population densities whose numbers are very difficult to reduce to an acceptable, non-damaging threshold, once established in the field. A high reproduction rate and/or a polyphagous lifestyle are key characteristics for the successful establishment and proliferation of PPN, usually placing root-knot nematodes (*Meloidogyne* spp.), cyst nematodes (*Globodera* spp. and *Heterodera* spp.), root-lesion nematodes (*Pratylenchus* spp.), the burrowing nematode (*R. similis*), and the stem and bulb nematode (*D. dipsaci*) as the most damaging for agricultural crops [11,12]. In forestry systems, *B. xylophilus* is uncontestably the most devastating [13].

Chemical control with synthetic nematicides is the most effective strategy to control PPNs, but due to their broad spectrum of activity, environmental toxicity, and considerable legislative pressure to restrict them, they are progressively being phased out, and the need for alternatives is pressing [14]. The integrated pest management of PPNs should, therefore, contemplate environmentally sound and economically sustainable control measures, and biological control agents (BCAs) are good candidates.

The concept of biological control is based on the idea of the direct or indirect exploitation of a pathogen or parasite’s natural enemies to inhibit or reduce the incidence or severity of a disease [15,16]. BCAs can be of different taxonomic origins: entomopathogenic nematodes, insect parasitoids, pathogens (bacteria, fungi, viruses), predators, protozoa, and weed-attacking herbivores [16,17]. Here, we consider BCAs organisms that are capable of suppressing nematodes, either by antagonism (being able to parasitize, kill, and consume their prey, or by producing molecules that negatively affect nematodes) or by providing plant-promoting effects and enhancing plant defenses against PPNs. Microbial biocontrol agents are often found and isolated from suppressive soils [18], which are usually defined as soils in which pathogens and parasites do not establish or persist, establish but cause limited or no disease, or establish and cause disease for a while, before subsiding [19]. However, a single management option rarely leads to the sustainable management of a nematode problem. Ideally, a successful nematode management strategy will involve the selection of a combination of options, provided they are applicable, appropriate, and economically viable [20].

Here, we focus on bacterial and fungal BCAs, analyzing data from 2018 to 2022, and review their potential to suppress some of the most important PPNs [11,12], specifically *A. besseyi*., *B. xylophilus*, *D. dipsaci*, *Globodera* and *Heterodera* spp., *Meloidogyne* spp., *N. aberrans*, *Pratylenchus* spp., *R. reniformis*, *R. similis*, and *X. index*. Lastly, we discuss the importance of promoting research on the biocontrol of PPNs and streamlining BCAs screening, and consider the future directions for this field.

## 2. Microbes against Plant-Parasitic Nematodes

**Figure 1:**
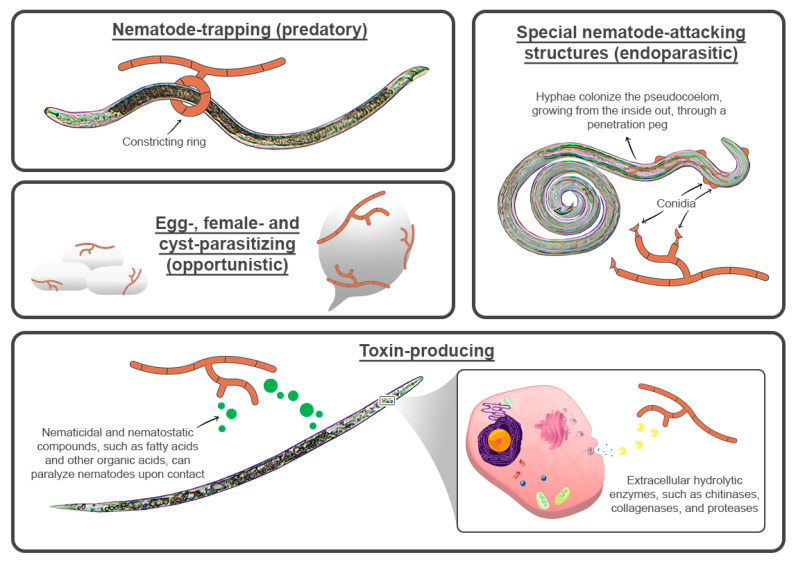
Illustrative representation of types and modes of action of nematophagous fungi.

Microbes developed a wide array of strategies to target both motile and sedentary PPNs life stages. Through specialized structures, such as constricting rings, three-dimensional hyphae networks, and adhesive spores, for example, predatory fungi can trap nematodes and prevent them from escaping [21,22] (Figure 1). Opportunistic saprotrophic fungi attack nonmotile stages, like eggs, cysts, and *Meloidogyne* females [23]. Endoparasitic fungi have developed specialized structures and strategies to feed on nematodes by luring them toward spores and forming a penetration peg upon contact, from which hyphae grow and colonize the pseudocoelom, resulting in the rupture of organs and tissues [24].

**Figure 2:**
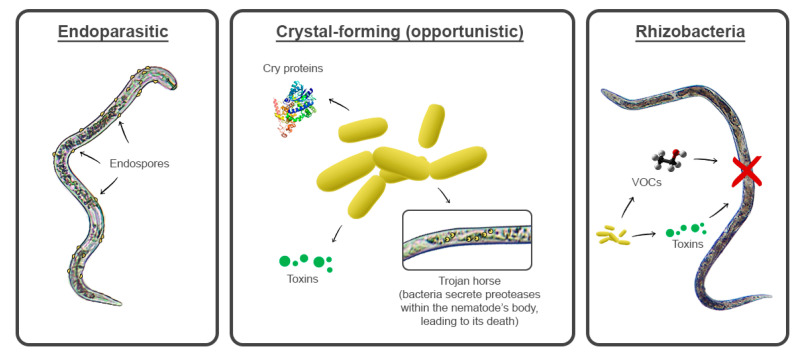
Illustrative representation of types and mechanisms of antagonism of bacteria on plant-parasitic nematodes.

Other microbes can produce and release nematicidal or nematostatic compounds into the soil [25], and mycotoxins are commonly employed by toxin-producing fungi to immobilize or kill nematodes [26,27]. However, not all microbial BCAs have suppressive effects on nematodes. Many bacteria and fungi (some of which are endophytes), including arbuscular mycorrhizal fungi (AMF), have plant-promoting effects instead and can induce plant defense mechanisms against PPNs, namely, by managing phytohormone levels, inducing signal substrate production, regulating gene expression, and enhancing protein production, and they have been extensively used as plant health promoters and BCAs against harmful nematodes [28,29,30,31,32,33,34,35,36,37]. Bacterial mechanisms to antagonize PPNs may include the production of antibiotics, endospores, hydrolytic enzymes, volatile organic compounds (VOCs), Cry proteins (pore-forming toxins), and Trojan horses, which lure nematodes by emitting VOCs and secreting proteases or toxins upon entry into their host, ultimately killing the nematode [17,38,39,40] (Figure 2).

Promising microbial BCAs, bacteria and fungi specifically, targeting the most important plant-parasitic nematodes are presented in Table 1 and Table 2, respectively.

### 2.1. Root-Knot Nematodes (RKNs), Meloidogyne spp.

RKNs are obligate parasites, with a widespread distribution across the globe, capable of feeding on almost every species of vascular plant. Their polyphagous lifestyle usually grants *Meloidogyne* spp. the title of the most damaging PPN. This genus consists of about 100 species as of 2021 [113], but the most important species, commonly referred to as the big four, are the tropical *M. arenaria*, *M. incognita*, and *M. javanica*, and the temperate *M. hapla*.

In the last 5 years, research on the management of RKNs has mostly focused on two species of the big four, namely *M. incognita* and *M. javanica*. Nevertheless, the emerging *M. enterolobii* and *M. graminicola* have also gained special attention. The current literature is especially dedicated to increasing knowledge on reducing or avoiding PPN infection in tomato, but other crops are also considered.

Among the main bacterial agents described for *Meloidogyne* spp., the genera *Bacillus*, *Pasteuria*, and *Pseudomonas*, followed by *Streptomyces* and some family Enterobacteriaceae members, have been the most analyzed. Liu et al. explored the drivers of the specificity change of *P. penetrans* on *M. arenaria* in peanut plots and crop rotations with peanut and soybean. Their results show a rapid change in the host specificity of *P. penetrans* against *M. arenaria*, both in space and time, and they observed an overall reduction in the attachment rate with samples from rotation plots relative to samples from peanut plots, which may reflect the lower abundance of the bacterial antagonist under crop rotation, potentially due to suppressed density of host nematodes [43]. Ghahremani et al. [31] studied the effects of *B. firmus* I-1582 on *M. incognita* and the root colonization of tomato and cucumber and noted that the bacterium degraded eggshells and colonized tomato roots more extensively than cucumber roots. The authors also observed that, although its optimal growth temperature is 35 °C, the bacterium was able to grow and form biofilms from 15 to 45 °C, while inducing systemic resistance in tomato but not in cucumber [31]. Indeed, salicylic acid (SA)- and jasmonic acid (JA)-related genes were primed at different times after nematode inoculation in tomato, but only the SA-related gene was upregulated at 7 days after nematode inoculation in cucumber [31]. Tian et al. [32] assessed the nematicidal activity of *B. velezensis* Bv-25 against *M. incognita* and its overall effects on cucumber and found that this strain inhibited egg hatching and produced a 100% mortality rate of J2s within 12 h of exposure to Bv-25 fermentation broth in vitro. Furthermore, Bv-25 colonized cucumber roots, effectively reducing the infection rate of J2s by 98.6% [32]. Pot trials showed that Bv-25 reduced cucumber root knots by 73.8%, and a field experiment demonstrated that the disease index was reduced by 61.6%, the cucumber height increased by 14.4%, and the yield increased by 36.5% in Bv-25-treated plants compared to the control [32]. Mazzuchelli et al. [41] examined two application methods of *B. subtilis* for the biological control of RKNs and root-lesion nematodes (RLNs) in sugarcane. Bacterial application at planting proved to be more effective in controlling both genera than applying *B. subtilis* post-emergence, and the effect was higher than that of carbofuran [41]. Engelbrecht et al. [42] reported that a filtrate mixture of *B. cereus*, *B. megaterium*, *B. subtilis*, and *B. thuringiensis* caused approximately 85–90% immobility of *M. enterolobii*, *M. incognita*, and *M. javanica* J2s after 96 h, theorizing that bioformulations with *Bacillus* spp. mixtures might be more effective than products from a single species in limiting juvenile motility. Bui et al. [45] found that bacterial volatiles emitted by *Bacillus* sp. and *Xanthomonas* sp. have the potential to control *M. graminicola*, albeit high volatile concentrations may hamper plant growth. Choi et al. [47] noted that the number of egg masses and root gall index produced by *M. incognita* were significantly curbed in the treatment group with two *Bacillus* strains, *B. thuringiensis* and *B. velezensis*, both in vitro and in planta, even when compared to the nematicide treatment. Interestingly, some strains showed host-specificity in their effects as biocontrol agents for RKNs [47]. Nasiou et al. [58] investigated the compatibility of fluazaindolizine and oxamyl with *P. penetrans* in populations of *M. incognita* and *M. javanica* and found that neither fluazaindolizine nor oxamyl had any negative effect on the rate of spore attachment. The spore-encumbered J2s were used to infect tomatoes, and RKN females without egg masses were extracted from the roots 50 days after inoculation and checked for eggs in the ovaries and mature spores of *P. penetrans* [58]. Although no mature endospores were present in the females, there was evidence of a low percentage of infection in a few treatments, which might be explained by a loss of the pathogenicity of the bacterium, as it kept in the form of dried roots for a long period [58]. *Pseudomonas fluorescens* CHA0 is capable of significantly reducing disease severity in tomato cultivars to a higher extent than in cucumber [61]. Other strains of *Bacillus* sp. CBSAL02 and *Pseudomonas* sp. CBSAL05 displayed broader activities, significantly reducing the hatching of *M. javanica* eggs by 74% and 54%, respectively [63]. Bhuiyan et al. [64] set up two experiments to determine the suppressive effects of *P. penetrans* endospores against *M. javanica* in sugarcane. In the first one, eggs of the RKN were inoculated into *Pasteuria*-free and naturally infested soils, and the results revealed that the RKN population was 96 and 99% lower in the naturally infested soil 19 and 37 weeks after inoculation, respectively [64]. The second experiment consisted of determining the effect of endospore concentration on the multiplication of *M. javanica*, and the results showed that regardless of harvest time, the severity of root galling and the number of nematode eggs produced per plant were inversely proportional to the endospore concentration [64]. Walia et al. [65] reported that olive plantation soils with a naturally high incidence of *P. penetrans* (50–90%) had suppressive levels and kept *M. javanica* populations below damaging thresholds.

When it comes to fungal BCAs of *Meloidogyne* spp., *P. lilacinum*, *P. chlamydosporia*, and *Trichoderma* spp. have been the most studied, followed by *A. oligospora*. Ghahremani et al. [34] studied the plant-dependent effects produced by *P. chlamydosporia* against *M. incognita*, both in cucumber and tomato, and found that two out of the five tested *P. chlamydosporia* isolates, M10.43.21 and M10.55.6, induced systemic resistance against the RKN in tomato but not in cucumber in split-root experiments. The M10.43.21 isolate reduced infection (32–43%), reproduction (44–59%), and female fecundity (14.7–27.6%), while M10.55.6 only reduced nematode reproduction (35–47.5%) in the two experiments [34]. Isolate M10.43.21 induced the expression of the SA pathway in tomato roots as early as 7 days after inoculation with the fungal isolate, and the JA signaling pathway was also upregulated at 7 days after nematode inoculation [34]. This demonstrates the differential ability of some isolates of *P. chlamydosporia* to induce systemic resistance against RKNs, although this appears to be plant-species dependent [34]. Pocurull et al. [36] conducted several experiments to determine the ability of two commercial *Trichoderma* formulations, *T. asperellum* T34 and *T. harzianum* T22, to induce systemic resistance in tomato and cucumber against *M. incognita*. The authors reported that both *Trichoderma* formulations induced resistance to *M. incognita* in tomato but not in cucumber [36]. T34 reduced the number of egg masses and eggs per plant by 71 and 54% in tomato, respectively, while T22 reduced 48% of the number of eggs per plant but not the number of egg masses [36]. Furthermore, T34 reduced the number of eggs per plant of the virulent *M. incognita* population in both resistant and susceptible tomato cultivars, irrespective of the suppressive soil, and its effect was additive with the *Mi-1.2* resistance gene [36]. Yan et al. [37] explored the suppressive effects of *T. harzianum* against *M. incognita* in tomato plants and observed that the fungus reduced the RKN infestation in 61.88%. While RKN infestation increased the levels of reactive oxygen species (ROS) and lipid peroxidation in tomato roots, colonization by *T. harzianum* significantly reduced the levels of ROS, malondialdehyde, and electrolyte leakage, and the activity of defense-related enzymes and the expression of associated genes significantly increased in plants treated with the fungus [37]. Moreover, *T. harzianum* inoculation prior to RKN infestation significantly increased the activity of pathogenesis-related proteins, while also increasing the levels of SA and JA [37]. Amarasinghe et al. [83] reported that *T. viride* significantly reduced the root galling of susceptible rice variety Bg 366 when compared to untreated plants. Tazi et al. [91] assessed the nematicidal potential of different fungal genera (*Arthrobotrys*, *Monacrosporium*, *Purpureocillium*, *Talaromyces*, and *Trichoderma*) in vitro and observed the highest RKN mortality rates after 72 h using *A. oligospora* and *P. lilacinum*. However, the same authors reported better results for the chemical control (abamectin) than the fungi tested in vivo [91]. Patil et al. [84] evaluated the efficacy of *P. lilacinum* and *T. viride* on *M. incognita* in cucumber and noted a significant reduction in nematode population with carbosulfan, followed by the highest dose of a liquid formulation with both fungi (15 mL/kg seed). The pathogenicity of 10 isolates of *Pochonia chlamydosporia* was compared by Vieira dos Santos et al. [87] and it varied between 38 and 65% against *M. incognita* eggs in vitro. The same study also found a strong relationship between rhizosphere colonization by the fungus and parasitism of RKN eggs [87]. Khan et al. [38] found that supplementing *P. chlamydosporia* with *Ageratum conyzoides* augmented the nematicidal effect of the fungus, suppressing root infestation caused by *M. incognita* while improving growth and the physiological attributes of chickpea. Similarly, Fayzia et al. [88] reported that *A. oligospora*, *P. lilacinus*, and the AMF *G. faciculatum* were effective in controlling *M. incognita* on cucumber in greenhouse conditions. Molinari and Leonetti [54] explored the induced resistance against *M. incognita* provided by a mixture of antagonistic fungi, *T. harzianum* TH 01 and *P. chlamydosporia* Pc50, with AMF, among other microorganisms, on tomato and found that the BCAs activated plant immunity and the fungi present in the formulation were indeed plant priming-inducers. Expósito et al. [82] noted that the combined use of *T. asperellum* T34 and beet molasses significantly reduced *Meloidogyne* spp. reproduction between 83 and 99%, compared to the single application of molasses, which varied between 49 and 99%. Kassam et al. [89] investigated the effect of the fungus *Metarhizium anisopliae* ITCC9014 on *M. incognita* in vitro and reported that 97 ± 2% of juveniles were parasitized after 3 days. Additionally, they noted that *M. anisopliae* ITCC9014 significantly reduced symptoms 40 days after inoculation, in terms of the total number of galls, females, egg masses, and eggs per egg masses, with no significant differences between the chemical nematicide carbofuran and the fungal treatment [89]. Lastly, an 82% reduction in the nematode multiplication factor was also observed [89].

### 2.2. Cyst Nematodes (CNs), Globodera, and Heterodera spp.

*Globodera* spp. are highly specialized, obligate endoparasitic nematodes and major quarantine pests, native to South America, having spread to nearly all potato-producing regions of the globe [114]. The major CN species are *G. pallida* and *G. rostochiensis* (potato cyst nematodes, PCNs), *Heterodera glycines* (soybean cyst nematode, SCN), *H. avenae*, *H. filipjevi* (cereal cyst nematodes, CCNs), and *H. schachtii* (beet cyst nematode, BCN), and CNs are known for their capacity to survive for prolonged periods in the soil in the absence of a suitable host [115,116], making cultural control through crop rotation or trap crops difficult and eradication, once established, nearly impossible. Although the economic impact of these PPNs is difficult to ascertain, *G. pallida* and *G. rostochiensis* might be responsible for worldwide potato crop losses of approximately 9% [117]. The SCN is the most devastating pest in soybean-producing areas throughout the United States and Canada [118], being responsible for economic losses ascending to USD 1.5 billion per year in the U.S. alone [119]. Crop losses caused by CCNs are heavily dependent on environmental conditions but can exceed 90% in some fields [120].

From 2018 to 2022, the most commonly studied bacterial agents against cyst nematodes belong to the *Bacillus* genus. Huang et al. [30] explored the effects of *B. firmus* I-1582 on the plant–nematode interaction between *A. thaliana* and *H. schachtii* and found that the root colonization by the rhizobacterium significantly protected *A. thaliana* from infestation by the BCN, negatively affecting nematode reproduction as well as pathogenicity and development over two generations in vitro [30]. Widianto et al. [66] evaluated the pathogenicity of *B. cereus*, *B. flexus*, *B. megaterium*, *B. pumilus*, and *B. subtilis* on *G. rostochiensis*, and noticed significantly contrasting protease and chitinase activities in these strains compared to the control. Ahmed et al. [68] investigated the effects of twenty *Bacillus* isolates on J2s of *H. avenae* in vitro, and significant mortality rates were observed for *B. cereus* XZ-33-3, followed by *B. cereus* XZ 24-2-1 and *B. weihenstephansis* MH-58-60-01. Out of all the tested *Bacillus* strains, *B. cereus* XZ 24-2-1, *B. cereus* XZ-33-3, *B. weihenstephansis* MH-58-60-01, and *B. thuringiensis* MH 032-003 fared the best in controlling *H. avenae* cyst development in greenhouse conditions [68]. In a subsequent study, Ahmed et al. [67] assessed the biocontrol potential of bacteria isolated from cysts against *H. avenae* in vitro. Morphological, physiological, and biochemical analyses showed that the most promising belonged to the *B. cereus* group, which was subjected to further testing under greenhouse conditions [67]. *Bacillus cereus* B48 was responsible for a 78% reduction in cyst development in roots, just below the avermectin control (84%) [67]. Zhao et al. [69] explored the biocontrol potential of bacterial strain *B. aryabhattai* Sneb517 against *H. glycines* and observed a 70% reduction in juveniles inside the roots and more than 60% in the number of cysts during field experiments. Lund et al. [70] assessed the efficacy of a bioformulation containing *P. nishizawae* against the SCN, under different management practices, and observed that the bacterium reduced the reproduction factor of *H. glycines* when the seeds were treated with the formulation.

In terms of fungal biocontrol, Vieira dos Santos et al. [87] performed in vitro bioassays to assess the parasitism of 10 isolates of *P. chlamydosporia* on *G. pallida* eggs, reporting pathogenicity varying between 34 and 49%. These low parasitism levels might be explained by the spontaneous hatching observed when *P. chlamydosporia* isolates seem to parasitize immature eggs more actively than eggs containing second-stage juveniles [87,121]. Zhang et al. [93] explored the effectiveness of *Beauveria bassiana* 08F04 and *Agrobacterium tumefaciens*-mediated transformants on *H. filipjevi* in vitro and observed significant changes in the growth rate and biocontrol potential among some of the transformants, particularly G10. They also noted that the application of wild-type *B. bassiana* 08F04 and transformant G10 significantly reduced the population of the CCN females in roots [93]. Benedetti et al. [94] tested the effect of the AMF *G. etunicatum* on *H. glycines* under greenhouse conditions and reported a 28% decrease in nematode females in the root system of mycorrhizal plants compared to untreated roots. These results suggest that *G. etunicatum* promotes tolerance of the host plant to the presence of the SCN [94].

### 2.3. Root-Lesion Nematodes (RLNs), Pratylenchus spp.

RLNs are obligate biotrophic, soil-inhabiting parasites recognized worldwide as major constraints to important agricultural crops, such as cash crops (cotton and coffee), food crops (cereals, fruits, and vegetables), fed crops (alfalfa), industrial crops (sugarcane), oil crops (soybean), and ornamental crops [122,123]. The motile stages of RLNs are able to enter and leave their host plant, feeding on root cells (epidermis, cortex, and vascular cylinder) and causing extensive necrotic lesions, eventually leading to cell death [123]. As a result, infected plant hosts often exhibit a decrease in root system development (distortion or stunting) and poor growth and yield. This situation is worsened by the fact that RLNs are also known to form disease complexes with other root pathogens, one such example being *P. penetrans* and *Verticillum dahlia* [124].

Controlling RLNs is a major challenge for crop producers. Thus far, a total of 103 *Pratylenchus* species have been described [125,126], which can be underestimated due to the low number of morphological features and high intraspecific variability that characterize them [122]. Hence, in the past five years, the study of biological control has been limited to a few RLN species (*P. brachyurus*, *P. coffee*, *P. penetrans*, *P. vulnus*, and *P. zeae*), with only four studies focusing on bacteria and seven on fungi (Table 1 and Table 2). Bacterial species from *Bacillus*, *Pseudomonas*, and *Streptomyces* were the most analyzed against *Pratylenchus* spp., while the most common fungal biocontrol agents were AMF (specifically from the *Glomus* genus) and *Trichoderma* spp. Promising results were obtained for *P. brachyurus* using different fungal species in corn and soybean [95,96,97,98]. Dias-Arieira et al. [97] compared the single application with the combined activity of *P. lilacinum* and *T. harzianum* in soybean crops, denoting that both fungi were more effective when applied independently. In a later study, different combinations of *B. subtilis*, *P. lilacinum*, and *T. asperellum* were tested against *P. brachyurus* infecting soybean. All combinations were efficient in controlling this RLN, outperforming the chemical nematicide abamectin, 120 days after sowing. The reproduction factor (Rf) of *P. brachyurus* was lower (Rf = 0.4) in the treatment combining *B. subtilis* and *P. lilacinum* in the crop season, while in the fallow season, the treatment with *P. lilacinum* alone resulted in the most significant reduction (Rf = 0.6) [98]. Pacheco et al. [96] showed that *P. chlamydosporia* Pc-3, Pc-10, Pc-35, and *Trichoderma* sp. T-10 were the most effective for the control of *P. brachyurus* in soybean and corn. Using an in vitro approach, Oliveira et al. [95] tested *Trichoderma* spp. extracts (non-volatile metabolites) against J2s of *P. brachryurus* and recorded 41–46% mortality rates with *T. asperellum* T00, and 64–65% with *T. harzianum* ALL42. Afterwards, these *Trichoderma* species were applied to two soybean cultivars commonly grown in Brazil (BRSGO Caiapônia and BRS 8560RR), under greenhouse conditions, reducing the nematode populations by 51 and 89% using T00 and ALL42, respectively [95].

As for *P. coffeae*, Asyiah et al. [72] used a bacterial consortium composed of endophytic *Bacillus* sp. and *Pseudomonas* sp. directly applied onto Robusta coffee (*Coffea canephora* A.) pots, which successfully suppressed nematode population in soil and roots by nearly 60–70%. Additionally, Duong et al. [73] tested direct in vitro nematicidal activity of different isolates of *Arthrobacter*, *Bacillus*, *Enterobacter*, *Herbaspirillum*, and *Pseudomonas*, among which *B. mycoides* CCBLR14 and other non-described isolates from the *B. cereus sensu lato* group, CCBLR15, CCBLR13, and CCBMTR4, were the most antagonistic to *P. coffeae*.

In the case of *P. penetrans*, Marin-Bruzos et al. [74] tested different species of *Pseudomonas* and *Streptomyces* in both in vitro and in planta conditions, using the host plant *Allium fistulosum* L. They found that the most effective was *P. donghuensis* P17, causing 87 ± 8% paralysis after 48 h. *Pratylenchus penetrans* antagonism was also tested in vitro with different strains of the fungus *Clonostachys rosea* [100], after which the most promising strains were applied in natural soil infested with *Pratylenchus* spp. and *Heterodera* spp., in wheat cultures. However, the authors noted that the nematode mortality observed in *C. rosea* culture filtrates was highly variable between strains [100]. Moreover, the difference in the in vitro antagonism assay against *P. penetrans* correlated with antagonism against *H. glycines*, suggesting a lack of host specificity in *C. rosea* [100]. Ceustermans et al. [99] tested several species of AMF individually and in a mix, in apple (*Malus domestica* cv. Golden delicious). A significant decrease in a *P. penetrans* population (97% reduction) was obtained in apple seedlings inoculated with a mix of indigenous AMF species (a mix of 13 species belonging to the Glomeraceae and Claroideoglomeraceae families), while the treatment with *Glomus intraradices* alone was responsible for a 68% decrease in the RLN population [99]. Using the same plant host, Noura et al. [101] evaluated the effect of the nematophagous fungi *Verticillium leptobactrum* HR1 against another RLN, *P. vulnus*, associated with three apple rootstocks (MM106, MM111, and Ba29). Both in vitro testing (with the highest mortality of 75%) and greenhouse experiments showed that *V. leptobactrum* was able to significantly reduce the *P. vulnus* population, either in the soil or in the roots of these rootstocks. 

Sankaranarayanan and Hari [102] reported the use of AMF (*G. fasciculatum* and *G. mosseae*) and antagonistic fungi *A. oligospora*, *P. lilacinum*, and *P. chlamydosporia* for *P. zeae* control in sugarcane. Among different treatments applied under greenhouse conditions, the most effective was the combined use of *A. oligospora* and *G. fasciculatum*, with a 77% reduction in the RLN population density [102].

### 2.4. Pinewood Nematode (PWN), Bursaphelenchus xylophilus

The PWN is believed to be native to North America [127]. It is a devastating migratory endoparasite of conifers, mostly *Pinus* spp., especially in Asia and Europe, where it causes pine wilt disease (PWD) to autochthonous trees [13,128]. Four elements come into play during PWD: the PWN, its insect vector (*Monochamus* spp.), a susceptible pine host, and Ophiostomatales fungi, which the nematode feeds upon during its mycophagous phase [129]. While the prevention and regular monitoring of the PWN and its insect vector are the most common strategies to manage PWD, dissemination can eventually occur. In Europe, the nematode was first reported in mainland Portugal, in 1999 [130], and despite the country’s herculean efforts to halt its spread, the PWN has found its way to Madeira Island and a Northwestern province of Spain [131,132].

Biocontrol options are very limited, especially when it comes to bacterial agents. Liu et al. [76] reported that two isolates of *E. coli*, M131 and M132, and one of *S. marcescens*, M44, showed significant nematicidal activity against the PWN in vitro after 12 h. The most promising fungal BCAs comprise two species of the *Esteya* genus: *E. vermicola* [133] and *E. floridanum* [108]. Currently, nine isolates of *Esteya* spp. are described and they are frequently associated with insects [106,134]. Two *Esteya* spp. isolates have been successfully used to suppress the PWN in Asian pines, like *P. densiflora*, but the efficiency of their application in other pine species remains unknown. The infective cycle of *E. vermicola* begins when the fungus attracts the nematodes towards the hyphae, where the spores adhere to the PWN cuticle. These conidia usually germinate within 18–24 h, causing death after the nematode’s organs and tissues are completely destroyed by a mass of hyphae, growing outward and producing more lunate conidia to begin the cycle anew [105]. *Esteya floridanum* was recently discovered, so its efficiency and infection mechanism are only just beginning to be unveiled [108]. The benefits of *Esteya* spp. have also been demonstrated in vivo, where the survival rate of *P. densiflora* infected by the PWN can range from 30–50%, over a time period of 3–6 years, when these fungi were used as a remedial effect and depending on the culture substrate employed [104]. Furthermore, when *P. thunbergii* trees were inoculated with spores of *Esteya* spp. prior to nematode infection, their survival rate was significantly higher [107]. *Esteya floridanum* was also shown to have a positive effect in controlling the PWN on *P. koraiensis* seedlings, although the fungus was only able to defer the death of the treated plants for 2–6 weeks [108]. Nevertheless, seedlings are usually more susceptible to pathogens and pests [135], which might explain the results obtained by Li et al. [108].

Zhang et al. [110] reported that the fungus *Volutella citrinella* GUCC2219 exhibited a predation rate of 33% on the PWN after 72 h in vitro, and a fermentation broth of GUCC2219 was able to produce a mortality rate of 100% after the same period and under the same conditions.

Other fungal antagonists of the PWN were recently reported: *Graphilbum* spp. and *Leptographium* spp., which significantly reduced the nematode’s population density compared to the *Botrytis cinerea* control [109].

### 2.5. Reniform Nematode (RN), Rotylenchulus reniformis

Among the few described species of reniform nematodes, *R. reniformis* gained notoriety as the most economically significant, most likely due to its widespread distribution [136]. *Rotylenchulus reniformis* is a semi-endoparasitic nematode, occurring most notably in tropical and subtropical regions, where it parasitizes a wide variety of crops, including cotton, vegetable crops, and several tropical fruit species [137,138,139,140,141].

In terms of biocontrol, Xiang et al. [77] showed that rhizobacteria strains, *B. mojavensis* Bmo3 and *B. velezensis* Bve2, significantly reduced the total numbers of *R. reniformis* eggs at 45 days after planting on soybean under controlled conditions, while Bmo3 also significantly increased plant biomass during the same timeframe. In soybean field trials, the strain Bmo3 significantly reduced *R. reniformis* eggs/g root at 45 days after planting and was statistically equivalent to the chemical nematicide abamectin [77]. Lira et al. [111] investigated the biocontrol potential of filtrates from *Fusarium inflexum*, *Thielavia terricola*, *T. longibrachiatum*, *T. brevicompactum*, *T. harzianum*, *Penicillium citrinum*, and two new *Penicillium* species, and reported promising nematicidal and hatch-inhibitory activities. These fungi caused nematode mortalities that ranged from 58 to 100% and only 5 to 20% of juveniles hatched in the in vitro tests. The same authors performed in vivo tests with coriander and cowpea and concluded that filtrates from the aforementioned fungi significantly reduced the number of egg masses and the reproduction factor of *R. reniformis* [111].

### 2.6. Fanleaf Virus Nematode (FVN), Xiphinema index

*Xiphinema* spp., also known as dagger nematodes, are considerably larger than most PPNs and are exclusively ectoparasites. Some species of *Xiphinema* are virus vectors and can transfer them to the plant host upon feeding [142,143]. For instance, *Xiphinema* spp. can cause the death of important crops by spreading viral mosaic and wilting diseases, thereby leading to significant economic losses [144]. *Xiphinema index* has gained particular attention because it vectors the *Grapevine fanleaf virus*, one of the most serious viruses of grapevine [145], but also due to its widespread distribution across the globe [143,146,147,148]. Although *X. index*’s most important hosts are grapevine and fig, it is known to parasitize other plants [149]. The nematode’s feeding activity causes poor root extension, resulting in swelling and gall formation, and leading to the reduced growth of infected plants.

Aballay et al. [78] explored the potential for the biocontrol of bioformulations containing different combinations of rhizobacterial agents on *X. index* on grapevine under greenhouse conditions. They showed that the powder formulation with *Brevibacterium frigoritolerans*, *B. megaterium*, *B. thuringiensis*, and *B. weihenstephanensis* was the most effective, which was comparable to the effect of the chemical nematicide Rugby® 200 CS (cadusafos) in suppressing the nematode [78]. On the other hand, Aballay et al. [78] noted that all the tested microbial agents and formulations, regardless of combination and type, decreased the severity of damage produced by *X. index*.

### 2.7. Other Economically Important PPNs

Between 2018 and 2022, biocontrol research on *R. similis*, *D. dipsaci*, *N. aberrans* and *A. besseyi* has been very limited, despite their economic relevance.

The burrowing nematode (BN), *R. similis*, is a polyphagous, migratory endoparasite, globally widespread but occurring mostly in tropical and subtropical regions, especially where bananas are grown. The BN can also be very destructive in citrus orchards and black pepper, among other horticultural crops [150]. The juvenile stages and adult females of *R. similis* are infective; contrastingly, males have an atrophied stylet and are non-parasitic to plants. Thammaiah et al. [75] combined two bacteria species, *B. subtilis* and *P. fluorescens*, with *P. lilacinus* to manage *R. similis* on banana. Both treatments were effective in nematode reduction, yet the best results were obtained when applying the chemical nematicide carbofuran [75].

Within the Anguinidae family, the stem and bulb nematode (SBN), *D. dipsaci*, is characterized by attacking a wide range of field crops, like broad bean, corn, garlic, onion, sugar beet, and ornamental plants, such as narcissus and tulips, to name a few [151]. This species is well-adapted to temperate conditions, specifically when humidity is adequate. *Ditylenchus dipsaci* is highly tolerant to desiccation, in contrast with other PPNs. While studying the interaction between two garlic pathogens, *D. dipsaci* and *Fusarium oxysporum* f. sp. *cepae*, McDonald et al. [103] unexpectedly reported that their combined effect was less severe in the bulb than when present separately. In fact, the inoculation of *F. oxysporum* after *D. dipsaci* reduced the disease severity index from 61.1 (combined application) to 8.3, suggesting either an antagonistic effect between both pathogens or a defensive response from the plant host [103]. Turatto et al. [63] described the reduced motility (>50%) of *Ditylenchus* spp. in vitro when inoculated with *Bacillus* sp. CBSAL02 and *Pseudomonas* sp. CBSAL05 strains.

*Nacobbus aberrans*, also known as the false root-knot nematode (FRK), produces galls that are similar in appearance to those caused by RKNs and is therefore often misdiagnosed based on symptoms alone. The FRK was originally described in the American continent and should be regarded as a species complex, due to the high molecular variability among populations and difference in host range. Wong-Villarreal et al. [80] and Méndez-Santiago et al. [79] reported the use of *Serratia ureilytica* and *Serratia* sp. as good candidates for the management of *N. aberrans*. Bernardo et al. [112] reported the efficacy of the arbuscular mycorrhizal fungus *Rhizophagus intraradices* B1 as a promising candidate for biocontrol.

*Aphelenchoides besseyi*, commonly referred to as the white tip nematode (WTN) due to the symptoms and disease it causes on rice [152], is a foliar nematode. Although it can parasitize other plants, various rice producers reported yield losses of up to 60% directly associated with *A. besseyi* in infested regions [153]. The WTN feeds both ecto- and endoparasitically on above-ground plant parts, and just like other species of the same genus, it is also mycophagous, which is crucial for survival in the absence of a suitable host. Phylogenetic data suggest that *A. besseyi* acquired plant parasitism relatively recently [154]. Recent studies on the biological control of *A. bessyi* are scarce, but Tülek et al. [81] reported that the independent application of the bacterial symbiont *Xenorhabdus bovienii* and *P. lilacinum* were effective in suppressing the WTN, theorizing that the concomitant use of both BCAs could yield better results. Zhang et al. [110] found that the fungus *Volutella citronella* GUCC2219 had a 59.45% predatory rate on the WTN and killed 100% of the nematodes after 72 h in vitro.

## 3. Future Prospects

Biological control, just like any other pest management method, has many benefits, but it is a direct intervention into the actual state of the ecosystem, and we need to be prepared for, or at least anticipate, potential hazards that may ensue from their application. Therefore, a thorough assessment of the benefits and risks should be undertaken before the use of BCAs, to provide stakeholders with the necessary information for efficient, safe, and sustainable pest control and production [155].

In general, many microbial strains and isolates are promising for the biological control of PPNs when tested in vitro, but not all provide consistent results when applied in field conditions, and, thus, not all can be developed into successful bionematicides. For example, the list of marketed plant growth-promoting rhizobacteria as BCAs towards PPNs is not extensive [156]. Likewise, not all, if any, BCAs may serve as stand-alone tools for the management of PPNs. On the other hand, microbial consortia are at the forefront of intensive research and hold great promise for biocontrol, but obstacles in the registration of BCAs may discourage commercial solutions for plant protection. Nevertheless, the number of augmentative biological control agents available from private markets seems to be increasing [157].

Prior to the period of this review, biocontrol studies were mostly descriptive, and some current ones still are. However, in recent years, the focus shifted to the molecular basis of nematode-microbe interactions. Unraveling such interactions on a molecular level is crucial for our understanding of how nematodes respond to BCAs, and vice versa, while taking into account how the plant host reacts to this interaction. Likewise, the identification and use of bacterial and fungal metabolites with nematicidal and nematostatic activity are also avenues of research of increasing interest worldwide [158,159,160,161,162]. This will open the door for optimized application methodologies, so we use BCAs to the best of our benefit. For that reason, as an ecologically based discipline, biological control should always consider an evolutionary perspective, incorporating the intrinsic genetic, phenotypic, and behavioral variation of BCAs and their targets, to fully comprehend the extent of these interactions [163,164,165]. Understandably, the increasing affordability and accessibility of DNA sequencing and the ability to genome-edit and design organisms with new characteristics are also paving the way for new possibilities for biological control.

Some genera of PPNs are consistently left out of the current biocontrol literature, despite the threat they pose to plant health and food security. Granted, the lack of standardized methods and the main focus on bacterial and fungal BCAs, to the detriment of other microorganisms, might also hinder other research avenues. Investigating neglected PPNs would open the door to the development of more targeted management strategies. Furthermore, a lot of recent studies are carried out in vitro and with single inoculants or by-products (filtrates and lysates), without providing realistic applications in natural conditions.

Regardless of how promising BCAs may be, they still face many challenges. While the mass production of microbial agents, storage, release methods, long-lasting, and potential adverse effects on non-target organisms remain some of the biggest constraints in the implementation of successful biocontrol strategies, the BCA’s biology may not be compatible with the application technique and formulation, or suitable to the environment where it would ideally be applied. Likewise, microbial BCAs can lose efficacy under adverse environmental conditions, and climate change may be detrimental to natural enemies and compromise their ability to control pests in otherwise temperate climate conditions, although these impacts are difficult to predict [166,167,168].

## Figures and Tables

**Table 1:** List of potential bacterial biocontrol agents against major plant-parasitic nematodes infesting agricultural and silvicultural systems (2018–present).

Major PPN	Biocontrol Agent(s)	Nematode Species	Plant Host	Reference
Root-knot nematodes (*Meloidogyne* spp.)	* Bacillus subtilis *	*Meloidogyne* spp.	Sugarcane	[41]
	*B. cereus*, *B. subtilis*, *B. thuringiensis*, *Priestia megaterium* (basionym: *B. megaterium*)		Soybean	[42]
	*Pasteuria penetrans*	*M. arenaria*	Peanut	[43]
	*Pseudomonas putida + Trichoderma harzianum*	*M. graminicola*	Rice	[44]
	*Bacillus* sp., *Paenibacillus* sp., *Xanthomonas* sp.			[45]
	*Brevundimonas* sp., *Microbacterium* spp.	*M. hapla*	–	[46]
	*Cytobacillus firmus* (basionym: *Bacillus firmus*)	*M. incognita*	Cucumber and tomato	[31]
	*Bacillus velezensis*		Cucumber	[32]
	*Bacillus thuringiensis*, *B. velezensis*		Tomato	[47]
	*Bacillus cereus*, *B. halotolerans*, *Cytobacilluskochii* (basionym: *B. kochii*), *Cytobacillus* *oceanisediminis* (basionym: *B. oceanisediminis*), *B.* *pseudomycoides*, *B. pumilus*, *B. toyonensis*, *Pseudomonas aeruginosa*			[48]
	*Brucella pseudogrignonensis* (basionym: *Ochrobactrum pseudogrignonense*)			[49]
	*Bacillus velezensis*		Cucumber	[50]
	* Streptomyces antibioticus *		Tomato	[51]
	* Paenibacillus alvei*, *Priestia aryabhattai* (basionym: *Bacillus aryabhattai*)		Tomato and carrot	[52]
	* Burkholderia arboris *		Tobacco	[53]
	* Agrobacterium radiobacter*, *Bacillus subtilis*, *Streptomyces* spp.		Tomato	[54]
	*Bacillus cereus*, *B. licheniformis*, *Lysinibacillus sphaericus*, *P. brassicacearum*, *P. fluorescens*			[55]
	* Serratia proteamaculans *			[56]
	*Bacillus cereus*, *Pseudomonas putida*		Patchouli	[57]
	* Pasteuria penetrans *		Tomato	[58]
	*Bacillus safensis*, *Lysinibacillus fusiformis*, *Priestia megaterium* (basionym: *B. megaterium*), *Pseudomonas resinovorans*, *Sphingobacterium daejeonense*	*M. javanica*	Tomato	[59]
	*Bacillus halotolerans*			[60]
	*Pseudomonas fluorescens*		Tomato and cucumber	[61]
	* Bacillus altitudinis *		Eggplant and cucumber	[62]
	* Bacillus * sp., *Pseudomonas* sp.		Garlic and soybean	[63]
	* Pasteuria penetrans *		Sugarcane	[64]
			Olive	[65]
			Tomato	[58]
Cyst nematodes (*Globodera* and *Heterodera* spp.)	*Bacillus cereus*, *B. pumilus*, *B. subtilis*, *Priestia flexa* (basionym: *B. flexus*), *P. megaterium* (basionym: *B. megaterium*)	*G. rostochiensis*	Potato	[66]
	*Bacillus* spp.	*H. avenae*	Wheat	[67]
	*Bacillus cereus*, *B. mycoides* (basionym: *B. weihenstephanensis*), *B. thuringiensis*			[68]
	*Priestia aryabhattai* (basionym: *Bacillus aryabhattai*)	*H. glycines*	Soybean	[69]
	*Pasteuria nishizawae*			[70]
	*Ensifer fredii* (basionym: *Sinorhizobium fredii*)			[71]
	*Cytobacillus firmus* (basionym: *Bacillus firmus*)	*H. schachtii*	*Arabidopsis thaliana*	[30]
Root lesion nematodes (*Pratylenchus* spp.)	*Bacillus subtilis*	*Pratylenchus* spp.	Sugarcane	[41]
	*Bacillus* spp., *Pseudomonas* sp.	*P. coffeae*	Coffee	[72]
	*Bacillus cereus sensu lato*, *B. mycoides*		–	[73]
	*Streptomyces microflavus* (basionym: *Streptomyces fulvissimus*), *S. venezuelae*, *S. anulatus*, *Pseudomonas donghuensis*, *Pseudomonas* sp.	*P. penetrans*	Onion	[74]
Burrowing nematode (*Radopholus similis*)	*Pseudomonas fluorescens + Purpureocillium lilacinum*	*R. similis*	Banana	[75]
	*Bacillus subtilis + Purpureocillium lilacinum*			
Stem and bulb nematode (*Ditylenchus dipsaci*)	*Bacillus* sp., *Pseudomonas* sp.	*Ditylenchus* spp.	Garlic	[63]
Pinewood nematode (*Bursaphelenchus xylophilus*)	*Escherichia coli*, *Serratia* sp.	*B. xylophilus*	–	[76]
Reniform nematode (*Rotylenchulus reniformis*)	*Bacillus mojavensis*, *B. velezensis*	*R. reniformis*	Soybean	[77]
Fanleaf virus nematode (*Xiphinema index*)	*Bacillus amyloliquefaciens*, *B. mycoides* (basionym: *B. weihenstephanensis*, *B. thuringiensis*, *Peribacillus frigoritolerans* (basionym: *Brevibacterium frigoritolerans*, *Priestia megaterium* (basionym: *B. megaterium*), *Pseudomonas fluorescens*	*X. index*	Grapevine	[78]
Fake root-knot nematode (*Nacobbus aberrans*)	*Serratia* sp.	*N. aberrans*	–	[79]
	*Serratia ureilytica*		Chili pepper	[80]
White tip nematode (*Aphelenchoides besseyi*)	*Xenorhabdus bovienii*	*A. besseyi*	Rice	[81]
	*Bacillus thuringiensis*			

**Table 2:** List of potential fungal biocontrol agents against major plant-parasitic nematodes infesting agricultural and silvicultural systems (2018–present).

Major PPN	Biocontrol Agent(s)	Nematode Species	Plant Host	Reference
Root-knot nematodes (*Meloidogyne* spp.)	*Trichoderma asperellum*	*Meloidogyne* spp.	Tomato	[82]
	*Trichoderma viride*	*M. graminicola*	Rice	[83]
	*Purpureocillium lilacinum*, *Trichoderma viride*	*M. incognita*	Cucumber	[84]
	*Trichoderma asperellum*, *T. harzianum*		Cucumber and tomato	[36]
	*Lecanicillium muscarium*		Tomato	[85]
	*Trichoderma harzianum*			[37]
	*Penicillium chrysogenum*		–	[86]
	*Pochonia chlamydosporia*		Tomato and cucumber	[34]
	*Pochonia chlamydosporia*		Tomato	[87]
	*Pochonia chlamydosporia*		Chickpea	[38]
	*Arthrobotrys oligospora*, *Glomus faciculatum*, *Purpureocillium lilacinum*		Cucumber	[88]
	*Glomus* spp., *G. mosseae*, *G. viscosum*, *Pochonia chlamydosporia*, *Trichoderma harzianum*		Tomato	[54]
	*Metarhizium anisopliae*			[89]
	*Purpureocillium lilacinum*	*M. incognita* and *M. javanica*		[90]
	*Arthrobotrys brochopaga*, *A. oligospora*, *Monacrosporium thaumasium*, *Purpureocillium lilacinum*, *Talaromyces assiutensis*, *Trichoderma asperellum*, *T. hamatum*, *T. harzianum*	*M. javanica*	Tomato	[91]
	*Pycnoporus sanguineus*			[92]
Cyst nematodes (*Globodera* and *Heterodera* spp.)	*Pochonia chlamydosporia*	*G. pallida*	Potato	[87]
	*Beauveria bassiana*	*H. filipjevi*	Wheat	[93]
	*Glomus etunicatum*	*H. glycines*	Soybean	[94]
Root lesion nematodes (*Pratylenchus* spp.)	*Trichoderma* spp.	*P. brachyurus*	Soybean	[95]
	*Pochonia chlamydosporia*		Soybean and corn	[96]
	*Purpureocillium lilacinum*, *Trichoderma harzianum*		Soybean	[97]
	*Trichoderma asperellum*			[98]
	*Acaulospora longula*, *Claroideoglomus claroideum*, *Glomus intraradices* and other unidentified AMF	*P. penetrans*	Apple	[99]
	*Clonostachys rosea*		Wheat	[100]
	*Verticillium leptobactrum*	*P. vulnus*	Apple	[101]
	*Arthrobotrys oligospora*, *Glomus fasciculatum*	*P. zeae*	Sugarcane	[102]
Burrowing nematode (*Radopholus similis*)	*Purpureocillium lilacinum + Pseudomonas fluorescens*	*R. similis*	Banana [1]	
	*Purpureocillium lilacinum + Bacillus subtilis*			
Stem and bulb nematode (*Ditylenchus dipsaci*)	*Fusarium oxysporum* f. sp. *cepae*	*D. dipsaci*	Garlic	[103]
Pinewood nematode (*Bursaphelenchus xylophilus*)	*Esteya vermicola*	*B. xylophilus*	*Pinus densiflora*	[104,105,106,107]
	*Esteya floridanum*		*Pinus koraiensis* and *Larix olgensis*	[108]
	*Leptographium* spp., *Leptographium terebrantis*, *Graphilbum* spp., *Ophiostoma ips*		–	[109]
	*Volutella citrinella*		–	[110]
Reniform nematode (*Rotylenchulus reniformis*)	*Fusarium inflexum*, *Thielavia terricola*, *Trichoderma brevicompactum*, *T. harzianum*, *T. longibrachiatum*, *Penicillium citrinum*	*R. reniformis*	Coriander and cowpea	[111]
Fake root-knot nematode (*Nacobbus aberrans*)	*Rhizophagus intraradices*	*N. aberrans*	Chili pepper	[112]
White tip nematode (*Aphelenchoides besseyi*)	*Purpureocillium lilacinum*	*A. besseyi*	Rice	[81]
	*Volutella citrinella*		–	[110]
